# The LPA1/ZEB1/miR-21-activation pathway regulates metastasis in basal breast cancer

**DOI:** 10.18632/oncotarget.3774

**Published:** 2015-04-27

**Authors:** Debashish Sahay, Raphael Leblanc, Thomas G. P. Grunewald, Srikant Ambatipudi, Johnny Ribeiro, Philippe Clézardin, Olivier Peyruchaud

**Affiliations:** ^1^ INSERM, UMR1033, UCB Lyon 1, Faculté de Médecine Lyon Est, Lyon, France; ^2^ Laboratory for Pediatric Sarcoma Biology, Institute of Pathology, LMU Munich, Munich, Germany; ^3^ Epigenetics Group, International Agency for Research on Cancer, Lyon, France

**Keywords:** lysophosphatidic acid, ZEB1, miR-21, breast cancer, metastasis

## Abstract

Lysophosphatidic acid (LPA) is a bioactive lipid promoting cancer metastasis. LPA activates a series of six G protein-coupled receptors (LPA_1-6_). While blockage of LPA_1_ *in vivo* inhibits breast carcinoma metastasis, down-stream genes mediating LPA-induced metastasis have not been yet identified. Herein we showed by analyzing publicly available expression data from 1488 human primary breast tumors that the gene encoding the transcription factor ZEB1 was the most correlated with *LPAR1* encoding LPA_1_. This correlation was most prominent in basal primary breast carcinomas and restricted to cell lines of basal subtypes. Functional experiments in three different basal cell lines revealed that LPA-induced ZEB1 expression was regulated by the LPA_1_/Phosphatidylinositol-3-Kinase (Pi3K) axis. DNA microarray and real-time PCR analyses further demonstrated that LPA up-regulated the oncomiR miR-21 through an LPA_1_/Pi3K/ZEB1-dependent mechanism. Strikingly, treatment with a mirVana miR-21 inhibitor, or silencing LPA_1_ or ZEB1 completely blocked LPA-induced cell migration *in vitro*, invasion and tumor cell bone colonization *in vivo*, which can be restored with a mirVana miR-21 mimic. Finally, high *LPAR1* expression in basal breast tumors predicted worse lung-metastasis-free survival. Collectively, our results elucidate a new molecular pathway driving LPA-induced metastasis, thus underscoring the therapeutic potential of targeting LPA_1_ in patients with basal breast carcinomas.

## INTRODUCTION

Lysophosphatidic acid (LPA) is a naturally occurring bioactive lipid promoting cancer progression and metastasis [1, 2]. LPA exhibits growth-factor-like activities by mobilizing multiple intracellular signaling pathways down-stream a series of six G protein-coupled receptors (LPA_1–6_) that mediate proliferation, motility, and survival of both normal and cancer cells [1, 2]. Pharmacological blockage of LPA receptors with the pan-LPA antagonist BrP-LPA inhibits human breast MDA-MB-231 cancer cell motility *in vitro* and promotes tumor regression associated with a decrease in blood vessel density surrounding the tumors in a mouse xenograft model, suggesting that LPA signaling may be a potential therapeutic target for patients with breast cancers [3]. Among the LPA receptors, LPA_1_ is up regulated in many types of primary tumors, and plays an important role in regulating cancer malignancy due to pro-oncogenic and pro-metastatic properties [4]. Studies showed that induction of LPA_1_ expression induces metastasis in breast and ovarian cancer cells [5, 6] and stimulates in the motility of human pancreatic cancer cells [7]. Using immune compromised mice we demonstrated that expression of this receptor confers a high propensity to human breast cancer cells to induce bone metastasis [8]. Stable knockdown of LPA_1_ expression (using small-hairpin RNAs or treatments with the LPA_1–3_ antagonist Ki16425) inhibited the progression of osteolytic bone metastases by impairing breast cancer cell proliferation, motility and cytokine secretions [8]. Pharmacological inhibition of LPA_1_ with Debio-0719 inhibits spontaneous metastatic dissemination of breast cancer cells independently of primary tumor proliferation and angiogenesis [9]. In a later stage of cancer progression Debio-0719 induces breast metastasis dormancy in a murine model [10]. Therefore, LPA_1_ is an attractive target. However the underlying mechanism and the genes involved in LPA_1_-mediated breast cancer metastatic dissemination remains to be determined.

Breast carcinomas are currently classified in four molecular subtypes (luminal A, luminal B, HER2-enriched, basal-like) based on gene expression patterns [11]. The basal-type subgroup also refers as triple negative breast cancers (TNBC). Kennecke and colleagues in their seminal work determined that TNBC metastasize predominantly to the brain and lungs [12]. TNBC constitute 10%-20% of all breast cancers [13] and 70% of women with metastatic TNBC have a survival rate of less than 5 years [14]. Absence of estrogen receptor, progesterone receptor, and HER2/neu makes TNBC insensitive to some of the most effective therapies available for breast cancer treatment including HER2-directed therapy and endocrine therapies urging the need for identification of new molecular targets in TNBC.

In this study we investigated the activated genes involved in LPA-mediated metastatic dissemination of breast cancers. Using both genetic and pharmacological approaches *in vitro* and *in vivo* we demonstrate that the pro-invasive activity of LPA in TNBC cells depends on the LPA_1_/Pi3K/ZEB1/miR-21 activation cascade. These findings identify LPA_1_ as a potential therapeutic target for patients with triple negative breast cancers.

## RESULTS

### *LPAR1* expression correlates with *ZEB1* in basal breast cancers

**Figure 1:**
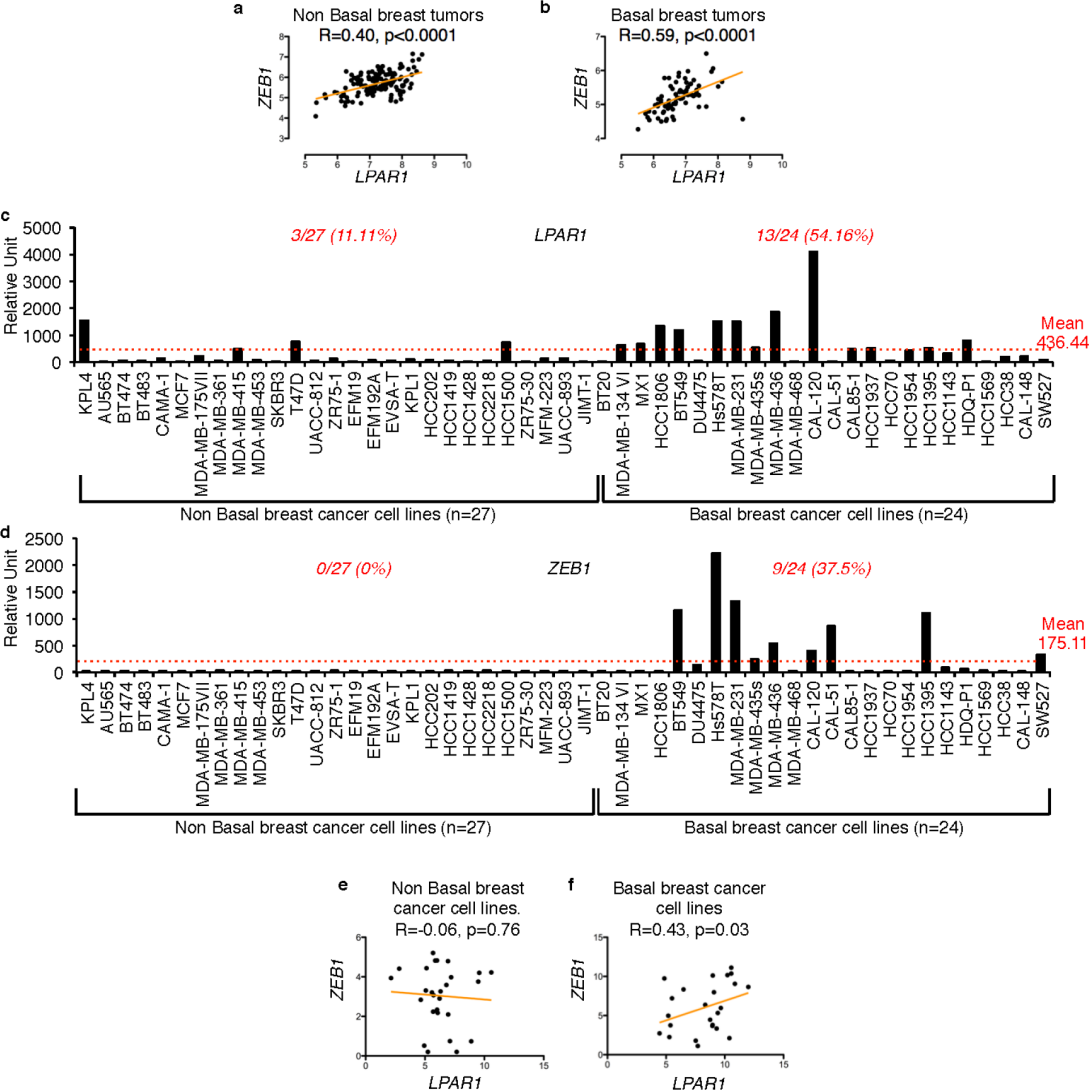
High correlation between *LPAR1* and *ZEB1* in basal breast cancer subtype Scatter plots for *LPAR1* and *ZEB1* gene correlation were constructed using data combined from three publically available databases (GSE20685, GSE21653, and GSE1456) of human primary breast tumors of **a.** non basal subtype (*n* = 138; r spearma *n* = 0.40) and **b.** basal subtype (*n* = 75, r spearma *n* = 0.59). **c.** *LPAR1* and d) *ZEB1* expression data from 51 breast cancer cell was extracted using BIOGPS online tool from GSE12777 data set, sub-classified into non basal (*n* = 27) and basal (*n* = 24) subtypes. The scatter plots are also shown for the correlation between *LPAR1* and *ZEB1* for both the e) non basal (*n* = 27; r spearman = −0.06) and **f.** basal (*n* = 24; r spearma *n* = 0.43) subtypes of human breast cancer cell lines.

In order to identify the genes correlated with *LPAR1*, we screened nine publicly available breast tumor databases (GSE2109; GSE5460; GSE1456; GSE2034; GSE12276; GSE3494; GSE2603; GSE7390; GSE16391) comprising of a total of 1488 patient samples. We found *ZEB1* to be the most correlated gene to *LPAR1* (Table 1). To further explore whether the correlation between *LPAR1* and *ZEB1* was specific to a particular subtype of breast cancer, we sub-grouped patient samples from 3 publicly available databases of primary breast tumor (GSE20685; GSE21653; GSE1456) into basal (*n* = 75) and non basal subtypes (*n* = 138) based on the ER, PR and HER2 receptor expression status. We found that the correlation between *LPAR1* and *ZEB1* was stronger in the basal subtype (R Spearma *n* = 0.59) of human primary breast tumors than the non-basal subtype (R Spearma *n* = 0.40) (Figures 1a and 1b). We then extracted the *LPAR1* and *ZEB1* expression data from 51 human breast cancer cell lines from the publically available database GSE12777, classified into basal (*n* = 24) and non basal subtypes (*n* = 27). We observed that 54.16% of the basal breast cancer cell lines expressed *LPAR1* and 37.5% expressed *ZEB1* above the overall means. In contrast, only 11.1% of the non basal breast cancer cell lines expressed *LPAR1* and none expressed *ZEB1* above the corresponding overall means (Figures 1c and 1d). Scatter plot analysis (Figures 1e and 1f) for *LPAR1* and *ZEB1* correlation in the non basal and basal breast cancer cell lines highlights the fact that *LPAR1* and *ZEB1* expression correlates significantly in the basal but not in the non-basal subtypes. We did not observe any positive correlation between *LPAR2* and *LPAR3* to *ZEB1* in either subtype (Supplementary Figures 1a, 1b, 1c, 1d, 1e and 1f).

**Table 1:** Highest positively correlated genes (Top50) to *LPAR1* in human primary breast tumors

Rank	Gene symbol	Coeff. of correlation	Gene name
**1**	ZEB1	**0,62178**	Zinc finger E Box-binding homeobox 1
**2**	TCF4	**0,58822**	Transcription factor 4
**3**	HTRA1	**0,58344**	HtrA serine peptidase 1
**4**	RECK	**0,56756**	reversion-inducing-cysteine-rich protein with kazal motifs
**5**	ZCCHC24	**0,55633**	zinc finger, CCHC domain containing 24
**6**	SPARCL1	**0,55544**	SPARC-like protein 1
**7**	SPON1	**0,54889**	Spondin 1
**8**	DCN	**0,54867**	Decorin
**9**	GLT8D2	**0,54800**	Glycosyltransferase 8 Domain Containing 2
**10**	ECM2	**0,54656**	Extracellular Matrix Protein 2
**11**	PDGFRL	**0,54478**	Platelet-Derived Growth Factor Receptor-Like
**12**	LRRC17	**0,54233**	Leucine rich repeat containing 17
**13**	EFEMP2	**0,54211**	EGF containing fibulin-like extracellular matrix protein 2
**14**	FBLN1	**0,53767**	Fibulin1
**15**	ZFPM2	**0,53689**	Zinc finger protein, FOG family member 2
**16**	OLFML1	**0,52800**	Olfactomedin-like 1
**17**	CFH	**0,52522**	Complement factor H
**18**	BICC1	**0,52456**	Bicaudal C homolog 1
**19**	KANK2	**0,52433**	KN motif and ankyrin repeat domains 2
**20**	PDGFC	**0,52278**	Platelet derived growth factor C
**21**	PLSCR4	**0,52189**	Phospholipid scramblase 4
**22**	FBN1	**0,52133**	Fibrillin 1
**23**	MMP2	**0,52089**	Matrix metallopeptidase 2
**24**	NDN	**0,52089**	Necdin, melanoma antigen (MAGE) family member
**25**	EDNRA	**0,51422**	Endothelin receptor type A
**26**	LDB2	**0,51378**	LIM domain binding 2
**27**	GAS7	**0,51322**	Growth arrest-specific 7
**28**	ZNF423	**0,51122**	Zinc finger protein 423
**29**	FSTL1	**0,50956**	Follistatin-like 1
**30**	CRISPLD2	**0,50467**	Cysteine-rich secretory protein LCCL domain containing 2
**31**	CTSK	**0,50222**	Cathepsin K
**32**	PCOLCE	**0,50022**	Procollagen C-endopeptidase enhancer
**33**	OMD	**0,49878**	Osteomodulin
**34**	SERPINF1	**0,49444**	Serpin peptidase inhibitor, clade F member 1
**35**	CDH11	**0,49089**	Cadherin 11, type 2, OB-cadherin (osteoblast)
**36**	ASPN	**0,48944**	Asporin
**37**	NAV3	**0,48933**	Neuron navigator 3
**38**	PMP22	**0,48733**	Peripheral myelin protein 22
**39**	TGFB3	**0,48611**	Transforming growth factor, beta 3
**40**	COPZ2	**0,48489**	Coatomer protein complex, subunit zeta 2
**41**	LAMA2	**0,47978**	Laminin, alpha 2
**42**	FERMT2	**0,47378**	Fermitin family member 2
**43**	PALLD	**0,47233**	Palladin, cytoskeletal associated protein
**44**	EHD2	**0,47211**	EH-domain containing 2
**45**	LUM	**0,46922**	Lumican
**46**	OLFML3	**0,46856**	Olfactomedin-like 3
**47**	PDGFRB	**0,46711**	Platelet-derived growth factor receptor, beta polypeptide
**48**	NID1	**0,46444**	Nidogen 1
**49**	THBS2	**0,46444**	Thrombospondin 2
**50**	ANGPTL2	**0,46411**	Angiopoietin-like 2

The table shows the top 50 highest positively correlated genes to *LPAR1* (204036_at) across 9 publically available breast tumor databases (GSE2109; GSE5460; GSE1456; GSE2034; GSE12276; GSE3494; GSE2603; GSE7390; GSE16391) comprising a total of 1488 samples. The list of correlated genes to *LPAR1* was extracted using R2 genomics analysis and visualization platform.

### Functional blockade of LPA_1_ inhibits ZEB1 expression *in vitro* and *in vivo*

**Figure 2:**
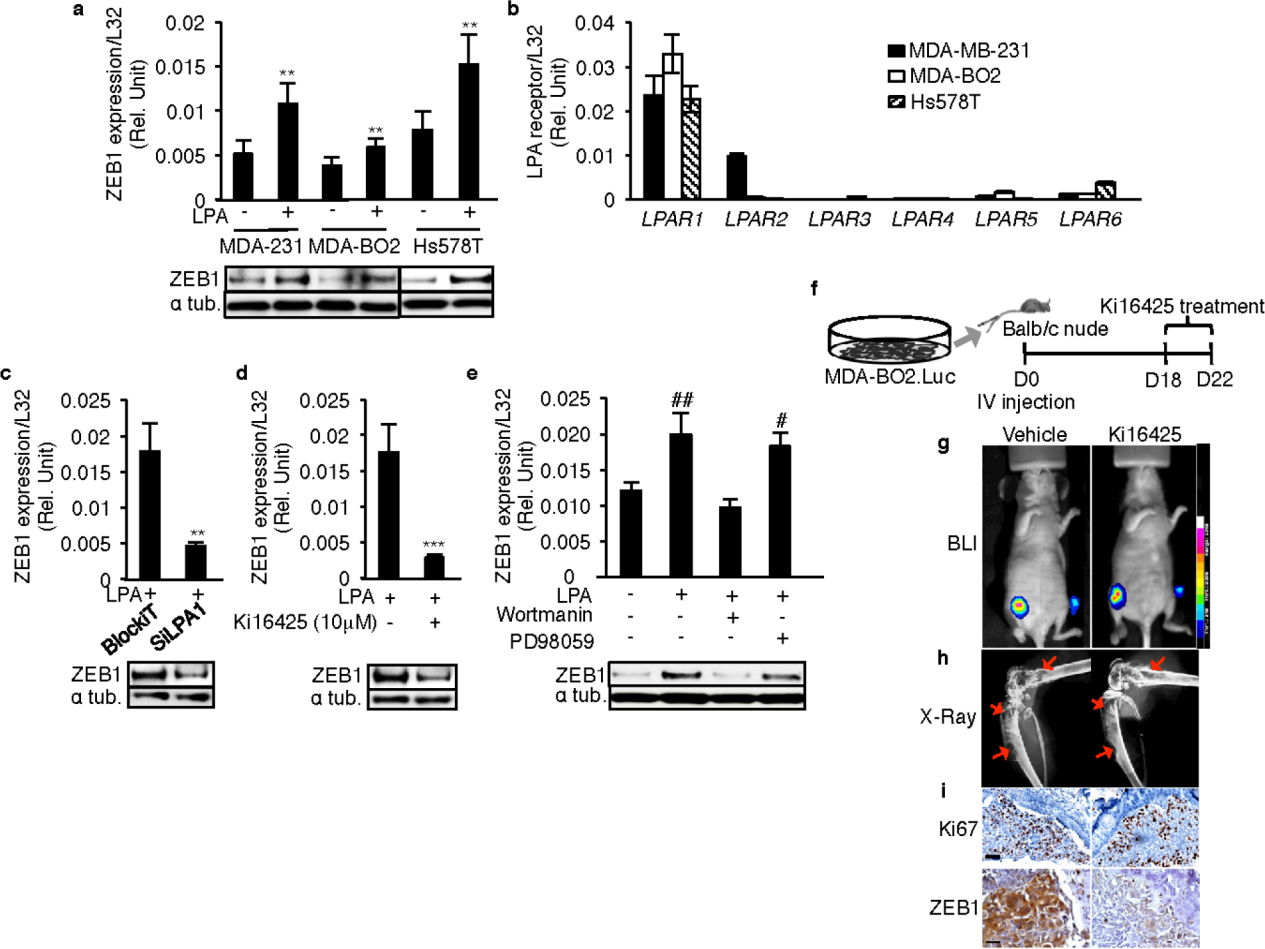
LPA_1_ mediates LPA-induced expression of ZEB1 *in vitro* and *in vivo* ZEB1 mRNA and protein levels were quantified in basal breast cancer cell lines **a.** MDA-231 (MDA-MB-231), MDA-BO2 and Hs578T basal human breast cancer cells on LPA (10 μM) stimulation. **, *p* < 0.01 vs correponding unstimulated cells using unpaired two-tailed student *t*-Test. **b.** Screening of LPA receptor expressions in MDA-MB-231, MDA-BO2 and Hs578T cells. ZEB1 mRNA and protein levels were quantified on MDA-MB-231 cells **c.** transfected with SiRNA against LPA_1_ and **d.** on treatment with Ki16425 (10 μM) in presence of LPA (10 μM) **, *p* < 0.01 vs BlockIT-transfected cells ; ***, *p* < 0.001 vs LPA stimulated MDA-MB-231 cells using unpaired two-tailed student *t*-Test. **e.** ZEB1 levels were quantified by Real time RT-PCR on treatment with PI3K inhibitor wortmannin or MEK1/2 inhibitor PD98059 in presence of LPA (10 **μM)** ZEB1 protein levels were also validated by Western blot. #, *p* < 0.05; ##, *p* < 0.01 vs unstimulated untreated 231 cells using one-way ANOVA with a Bonferroni pos*t*-test. **f.** MDA-BO2.luc cells were injected intravenously in Balb/c nude mice. The hind limbs were collected after 5 days of Ki16425 (25 mg/kg) treatment since the osteolytic lesions were observed by BLI (Day18). Images of **g.** BLI and **h.** X-Ray are shown from the vehicle treated and the Ki16425 treated group. **i.** Images of immunohistochemistry performed for Ki67 and ZEB1 are shown. The staining was done on 5 μM FFPE sections of the hind limbs collected from vehicle treated and Ki16425 treated groups. All values for results shown in panels A, B, C, D, I were the mean ± SD of 3 experiments.

We chose the human basal breast cancer cell lines MDA-MB-231, MDA-BO2 and Hs578T to analyze the role of LPA and LPA_1_ on ZEB1 expression *in vitro* and *in vivo*. Stimulation with LPA caused significant up-regulation of ZEB1 mRNA and protein levels in all three cell lines (Figures 2a). LPA receptor expression screening showed that LPA_1_ was predominantly expressed in all the three cell lines (Figure 2b). While LPA_2_ was also expressed in MDA-MB-231 cells, all three cell lines had very low or undetectable levels of LPA_3–6_ (Figure 2b). To further validate the relationship between LPA_1_ and ZEB1 we used MDA-MB-231 cells, as they co-express both LPA_1_ and LPA_2_. Blocking LPA_1_ expression by transfecting these cells with siRNA specifically directed against LPA_1_ (Supplementary Figure 2a) as well as by treatment with the pharmacological antagonist of LPA_1/3_, Ki16425 [15] inhibited the LPA-induced ZEB1 expression at both mRNA and protein levels (Figures 2c and 2d).

To assess the effect of antagonizing LPA_1_ on ZEB1 expression *in vivo*, we used an animal model of pre-established bone metastases caused by MDA-MB/B02.Luc cells. A short treatment period (daily administration for 5 days) of Ki16425 from day 18 post-tumor cell injection (Figure 2f), had no effect on the extent of bone metastasis lesions detected by BLI (Figure 2g) and X-ray (Figure 2h) analyses. Immunohistochemistry analyses performed on the bone sections from tumor bearing hind limbs revealed no significant difference of Ki67 staining in Ki16425-treated group compared to vehicle-treated group, indicating that the agent had no effect on cell proliferation. In contrast, the signal intensity of ZEB1 staining in the Ki16425 group was markedly lower than that of the vehicle group (Figure 2i), suggesting that inhibition of LPA_1_ activity *in vivo* inhibits ZEB1 expression at the site of bone metastases.

To identify the signaling pathway regulating the LPA/LPA_1_-induced ZEB1 expression, we treated MDA-MB-231 cells with the Pi3K inhibitor wortmannin (1 μM) or the MEK1/2 inhibitor PD98059 (100 μM). Only wortmannin treatment was able to inhibit LPA-induced up-regulation of ZEB1 in these cells, suggesting that the LPA/LPA_1_-induced ZEB1 expression was mediated through a Pi3K-dependent signaling pathway (Figure 2e).

### LPA up regulates microRNA miR-21 through a LPA_1_–dependent mechanism

**Figure 3:**
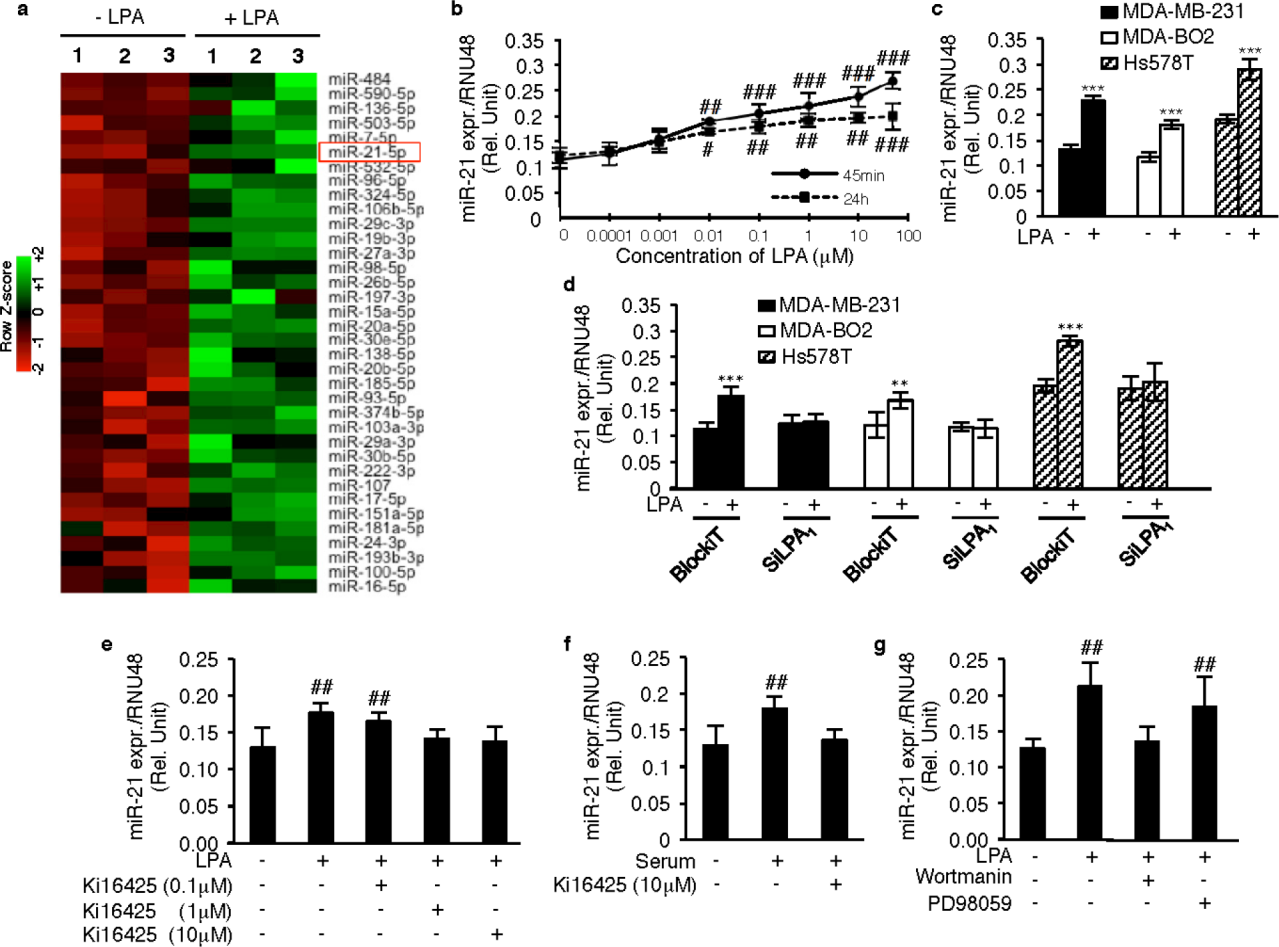
LPA_1_ regulates LPA-induced miR-21 expression **a.** Heat map was constructed for the upregulated miRNA's in MDA-MB-231 cells on LPA stimulation (1 μM) **b.** miR-21 expression was quantified using MDA-MB-231 on stimulation with an increasing dose of LPA (0.0001 **μ**M to 50 μM) at 45min and 24 h respectively. #, *p* < 0.05; ##, *p* < 0.01; ###, *p* < 0.001 vs Unstimulated 231 cells using one-way ANOVA with a Bonferroni pos*t*-test. **c.** qRT-PCR analysis of miR-21 was performed on MDA-MB-231, MDA-BO2 and Hs578T on LPA stimulation (10 μM) ***, *p* < 0.001 vs unstimulated 231/BO2 cells using unpaired two-tailed student *t*-Test. **d.** Relative expression of miR-21 was quantified by Real time RT-PCR by performing transient transfection with SiRNA against LPA_1_, and BlockiT (control SiRNA) on MDA-MB-231, MDA-BO2 and Hs578T cells on LPA (10 μM) stimulation. **, *p* < 0.01 vs BlockiT transfected unstimulated 231/BO2/Hs578T cells using unpaired two-tailed student *t*-Test. **e.** miR-21 relative expression was quantified in MDA-BO2 cells on treatment with different doses (0.1 μM, 1 μM, 10 μM) of Ki16425 (LPA_1_ and LPA_3_ antagonist) in presence of LPA (10 μM) or **f.** in presence of serum. ##, *p* < 0.01 vs Unstimulated BO2 cells using one-way ANOVA with a Bonferroni pos*t*-test; **, *p* < 0.01 vs unstimulated BO2 cells using unpaired two-tailed student *t*-Test. **g.** miR-21 levels were quantified by Real time RT-PCR on treatment with PI3K inhibitor (wortmannin) or MEK1/2 inhibitor (PD98059) in presence of LPA (10 μM) ##, *p* < 0.01 vs unstimulated untreated 231 cells using one-way ANOVA with a Bonferroni pos*t*-test. All values for results shown in panels B, C, D, E, F, G were the mean ± SD of 3 experiments.

In order to further identify the genes involved in the metastatic properties of LPA, we carried out a microarray profiling of miRNAs in MDA-MB-231 cells in response to LPA stimulation (GSE64100). Micro-RNA miR-21 was found to be one of the most up-regulated miRNAs in these cells following LPA stimulation (Figure 3a). MicroRNA miR-21 is known to act as an oncomiR, promoting metastasis in a large number of cancer types [16, 17]. Using the TaqMan RT-QPCR system, we confirmed the up-regulation of miR-21 by LPA in MDA-MB-231 cells and determined that LPA induced a dose-dependent increase in miR-21 expression by three to four folds after 45 min exposure (EC50 = 0.1 μM) that is sustained for at least 24 h (Figure 3b). In subsequent *in vitro* studies, cells were treated with LPA (10 μM) for 45min to reflect early exposure times of cells to LPA during the metastatic process. First, we observed that up regulation of miR-21 by LPA was a common feature between all the three basal human breast cancer cell lines, MDA-MB-231, MDA-B02 and Hs578T, (Figure 3c). As LPA_1_ was the predominant LPA receptor expressed by these cells, we silenced LPA_1_ expression by transient transfection of siLPA_1_. First, we confirmed that silencing LPA_1_ had no impact on the expression of the other LPA receptors (Supplementary Figure 2a, 2b, 2c). Intriguingly silencing LPA_1_ completely abolished LPA-induced miR-21 expression in all three cell lines (Figure 3d). Moreover, the treatment of the cells with Ki16425 inhibited LPA-induced miR-21 expression (Figure 3e). LPA is a constituent of the serum [18] that mediates tumor cells proliferation and invasion [8]. We found that Ki16425 treatment also inhibited miR-21 expression induced by serum in MDA-MB-231 cells (Figure 3f). To identify the signaling pathways regulating the *LPAR1*/miR-21 axis we used the same strategy as previously by treating the MDA-MB-231 cells with wortmannin or PD98059. Again, only wortmannin was able to inhibit LPA-induced miR-21 expression (Figure 3g), suggesting that LPA up-regulated miR-21 through an LPA_1_/PI3K-dependent mechanism.

### LPA-induced ZEB1 expression up regulates miR-21 in human primary breast tumors

**Figure 4:**
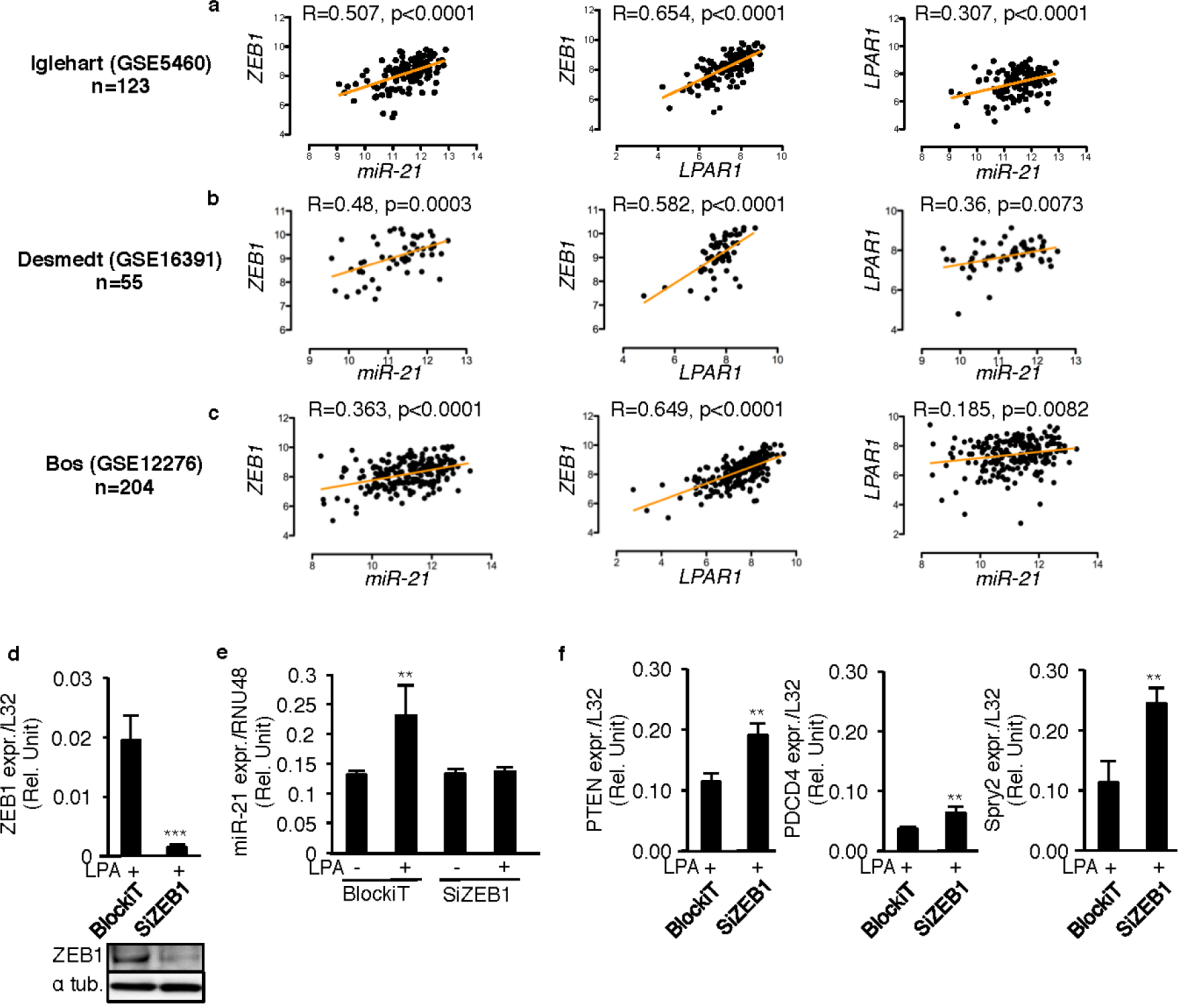
ZEB1 controls LPA-induced miR-21 expression through LPA_1_ activity Scatter plots of *miR-21* expression and *LPAR1* expression; *miR-21* expression and *ZEB1* expression; *LPAR1* expression and *ZEB1* expression were constructed with the Log2 tranformed values extracted from three publically available databases **a.** GSE5460, **b.** GSE16391, **c.** GSE12276 using R2 genomics analysis and visualization platform. Analysis of correlation and computation of linear regression of the data were performed using Prism v5.0b (GraphPad Software, Inc.). *p* < 0.05 were considered significant. MDA-MB-231 cells were transiently transfected with synthetic SiRNA's against ZEB1 (SiZEB1) and the control SiRNA (BlockiT) in presence of LPA (10 μM) **d.** ZEB1 levels were quantified to validate the efficacy of the SiZEB1. ***, *p* < 0.001 vs BlockIT transfected LPA stimulated cells using unpaired two-tailed student *t*-Test. **e.** miR-21 levels were quantified by taqman real time RT-PCR. **, *p* < 0.01 vs BlockIT transfected unstimulated 231 cells using unpaired two-tailed student *t*-Test. **f.** The mRNA levels of the target genes of miR-21 (PTEN, PDCD4, SPRY2) were quantified in the SiZEB1 and the BlockiT transfected MDA-MB-231 cells only on LPA (10 μM) stimulation. **, *p* < 0.01 vs BlockIT transfected LPA stimulated 231 cells using unpaired two-tailed student *t*-Test. All values for results shown in panels D, E, F were the mean ± SD of 3 experiments.

As LPA mediated up regulation of both ZEB1 and miR-21 through a common LPA_1_/PI3K axis, and because the strong correlation between ZEB1 and *LPAR1,* we examined the correlation between *LPAR1, ZEB1* and miR-21 in human primary breast tumors. *LPAR1/miR-21*, *ZEB1/miR-21* and *LPAR1/ZEB1* pairwise correlations were determined using data from in three publically available human breast cancer databases containing both mRNA and microRNA data (GSE5460; GSE16391; GSE12276). We found that *ZEB1/miR-21*, *LPAR1/ZEB1* and *LPAR1/miR-21* correlated significantly in all three databases (Figures 4a–4c).

To examine the direct link between LPA, ZEB1 and miR-21 expression, MDA-MB-231 cells were transfected with synthetic siRNAs against ZEB1 (Figure 4d). Silencing ZEB1 caused complete inhibition of LPA-induced miR-21 expression (Figure 4e) resulting in a significant increase in the mRNA levels of known miR-21 target genes *PTEN*, *PDCD4* and *SPRY2* (Figure 4f), suggesting that LPA might functionally impact on miR-21 activity through ZEB1.

**Figure 5:**
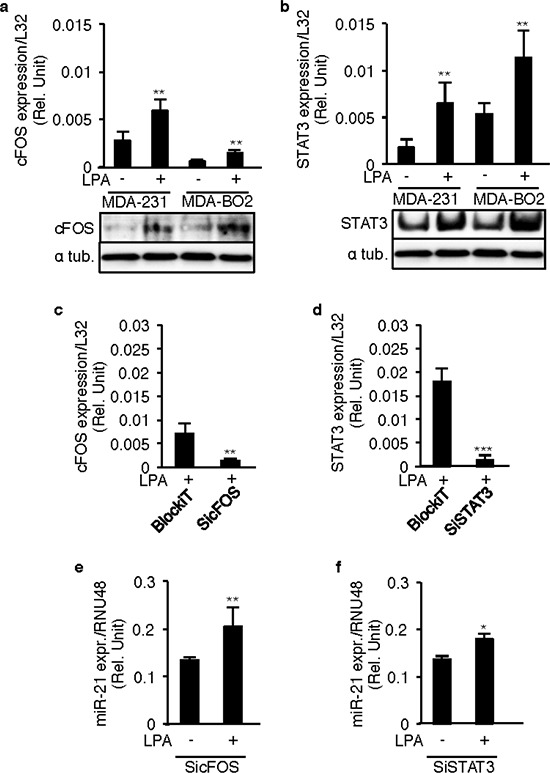
LPA-induced miR-21 expression is independent of STAT3 and cFOS **a.** cFOS, **b.** STAT3 mRNA and protein levels were quantified in MDA-MB-231 and MDA-BO2 cells by RT-QPCR on LPA (10 μM) stimulation. MDA-MB-231 cells were transiently transfected with synthetic SiRNA's against cFOS (SicFOS), STAT3 (SiSTAT3) and the control SiRNA (BlockiT) in presence of LPA (10 μM) and the **c.** cFOS and **d.** STAT3 levels were quantified to validate the efficacy of the SiRNA's. **e, f.** miR-21 levels were quantified in these SicFOS and SiSTAT3 transfected cells by taqman real time RT-PCR. *, *p* < 0.05, **, *p* < 0.01 ***, *p* < 0.001 using unpaired two-tailed student *t*-Test. All values were the mean ± SD of 3 independent experiments.

LPA is known for activating different transcription factors, including cFos and STAT3, in different cell lines [19, 20], and this was confirmed in MDA-MB-231 and MDA-B02 cells (Figure 5a and 5b). Because these factors also are regulators of miR-21 expression we evaluated whether cFos and STAT3 could mediate LPA-induced miR-21 expression. Silencing cFOS or STAT3 using specific siRNAs in MDA-MB-231 cells (Figures 5c and 5d) did not alter LPA-induced miR-21 expression (Figures 5e and 5f).

Altogether these results indicate that LPA induces miR-21 expression through an LPA_1_/PI3K/ZEB1-dependent pathway.

### LPA_1_ and ZEB1 mediate LPA-induced basal breast cancer migration and invasion in a miR-21-dependent manner

**Figure 6:**
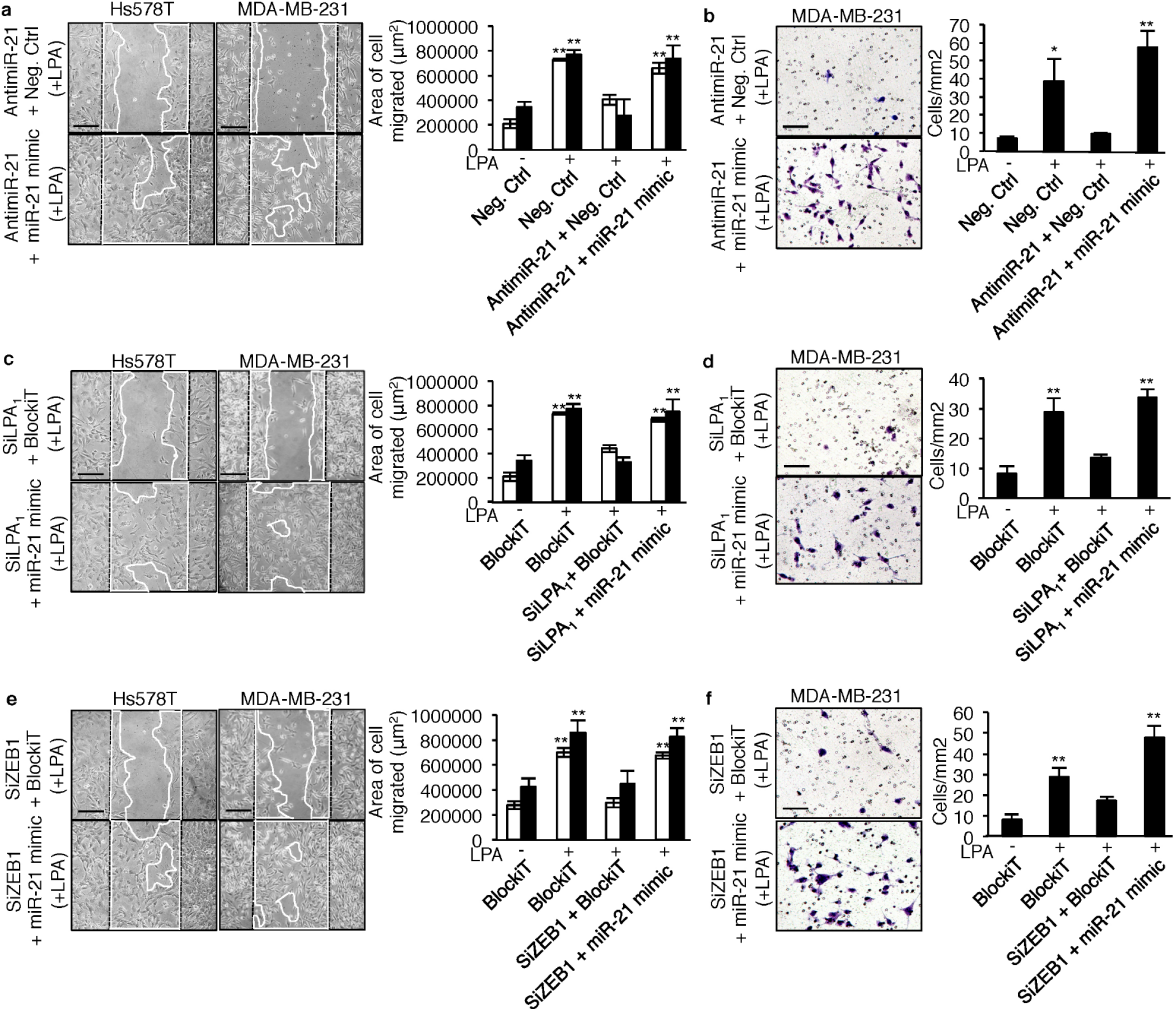
LPA-induced cell migration and invasion depend on miR-21 activity down-stream activation of LPA_1_ and ZEB1 Wound healing assay was performed on MDA-MB-231 (▀) and Hs578T (▀)cells transfected with **a.** negative control antimiR, antimiR-21+negative control antimiR and antimiR-21+miR-21 mimic; **c.** BlockiT, SiLPA_1_+BlockiT and SILPA_1_+miR-21 mimic and **e.** BlockiT, SiZEB1+BlockiT and SIZEB1+miR-21 mimic on LPA (1 μM) stimulation for which the representative images and the quantification of the area of cell migrated shown are at 24 h (for MDA-MB-231) and 48 h (for Hs578T) after wounding. The bar represents 200 μM. All values were the mean ± SD of 3 experiments. **, *p* < 0.01 vs unstimilated Neg.Ctrl AntimiR or BlockiT transfected MDA-MB-231 or Hs578T cells using one-way ANOVA with a Bonferroni pos*t*-test. For matrigel invasion assay 7.5 × 10^4^ MDA-MB-231 cells were transfected with **b.** negative control antimiR, antimiR-21+negative control antimiR and antimiR-21+miR-21 mimic; **d.** BlockiT, SiLPA_1_+BlockiT and SILPA_1_+miR-21 mimic; **f.** BlockiT, SiZEB1+BlockiT and SiZEB1+miR-21 mimic and were seeded on matrigel coated transwells and incubated for 24 h with LPA (1 μM) as chemo-attractant. Cell were fixed and stained with methanol and crystal violet and counted and photographed from 9 random fields from one insert. The representative images are shown. The bar represents 100 μM. All values were the mean ± SD of 3 experiments. *, *p* < 0.05; **, *p* < 0.01; ***, *p* < 0.001 vs Neg.Ctrl AntimiR or BlockiT using one-way ANOVA with a Bonferroni pos *t*-test.

To examine the functional impact of the LPA_1_/ZEB1/miR-21 pathway on the metastatic activity of LPA on basal breast cancer cells we carried out wound healing migration assays and matrigel invasion assays. Transfecting MDA-MB-231 and Hs578T cells with antimiR-21, SiLPA1 or SiZEB1 alone impaired LPA-induced cell migration (Figure 6a, 6c, 6e) and invasion (Figure 6b, 6d, 6f) as compared to the cells transfected with the Negative Control (antimiR control) or BlockiT (SiRNA control). Remarkably, in all cases, impaired motility and invasion was rescued when cells were co-transfected with a mirVana miR-21 mimic. This suggested that the role of LPA in these metastatic processes was mediated by miR-21 through the expression of ZEB1, down-stream activation of LPA_1_.

### Basal breast cancer cell bone colonization depends on miR-21 downstream ZEB1 activation

**Figure 7:**
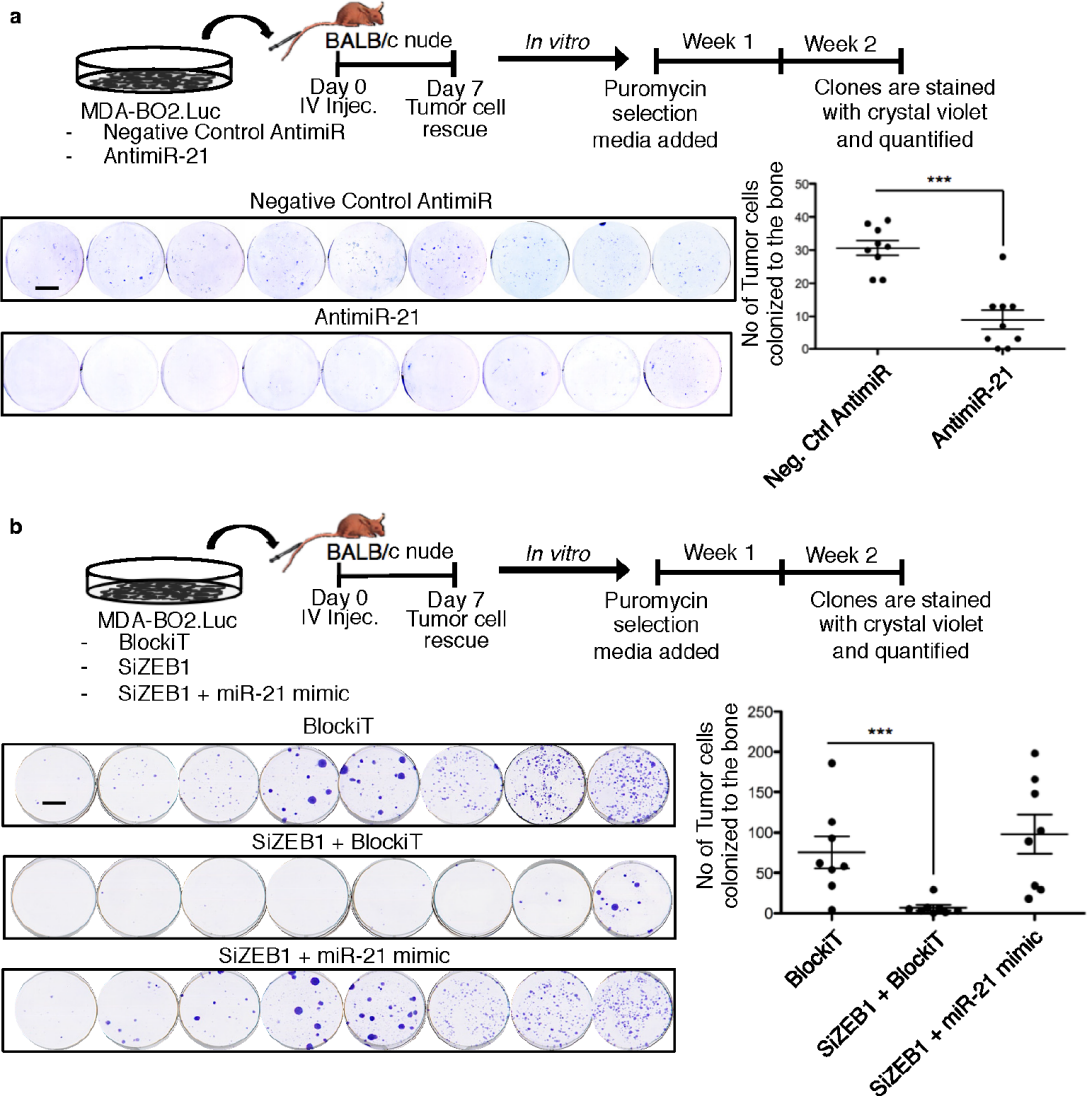
ZEB1 controls miR-21-dependent tumor cell bone colonization Tumor bone colonization experiment was performed using MDA-BO2.Luc cells transfected with **a.** Negative control antimiR or AntimiR-21; **b.** BlockiT or SiZEB1+BlockiT or SiZEB1+miR-21 mimic. The schema of the experiments are shown in the respective panels. The tranfected cells were injected intravenously through the tail vein. The picture of each well shown represents the bone marrow cells collected from one mice and cultured for 2 weeks in the presence of puromycin (1 μg/ml). Colonies corresponding to tumor cells that colonized to bone (TBC) were fixed and stained with 20% methanol-crystal violet (v/v) (scale bar: 1 cm). TBC colonies were counted. Results are expressed as mean of TBC (± S.E.M). ***, *p* < 0.001 versus mice injected with Neg.Ctrl AntimiR or Blockit transfected cells using two-tailed Mann Whitney test.

Micrometastasis formation is a well-recognized poor prognostic marker in breast cancers [21]. Patients with tripe negative metastasize to bone and with a higher incidence to lungs and brain [12]. To examine the role of miR-21 during the early phase of breast cancer cell metastasis we used our mouse model of tumor cell colonization to bone (TCB) in which MDA-BO2.luc cells transfected with AntimiR-21 or Negative control were injected intravenously BALB/c nude mice. Cancer cells that colonized the bone marrow cavity were collected seven days post injection and expanded *in vitro* for two weeks in the presence of puromycin that kills non-tumoral cells. Counting of tumor clones revealed that silencing miR-21 decreased TCB clone number by 70% compared to control (*p* < 0.001). This result indicated that miR-21 drives basal breast cancer cell metastasis to bone (Figure 7a).

We next addressed the functional relationship between miR-21 and ZEB1 *in vivo* using the TCB mouse model. Mice were injected with MDA-BO2.luc cells transfected with BlockiT (control SiRNA) or SiZEB1+BlockiT or SiZEB1+miR-21 mimic. Silencing ZEB1 significantly impaired the TCB, as judged by a significantly decrease by 88% (*p* < 0.001) in the number of TCB clones compared to the control group. Interestingly, the inhibition in the TCB induced by silencing ZEB1 was abolished when the cells were previously co-transfected with miR-21 mimic. The number of TCB clones in the groups of mice injected with breast cancer cells transfected with SiZEB1+miR-21 mimic was not significantly different to those obtained with cells transfected with BlockiT (Figure 7b). These results indicated that ZEB1 regulates the TCB in a miR-21-dependent manner.

**Figure 8:**
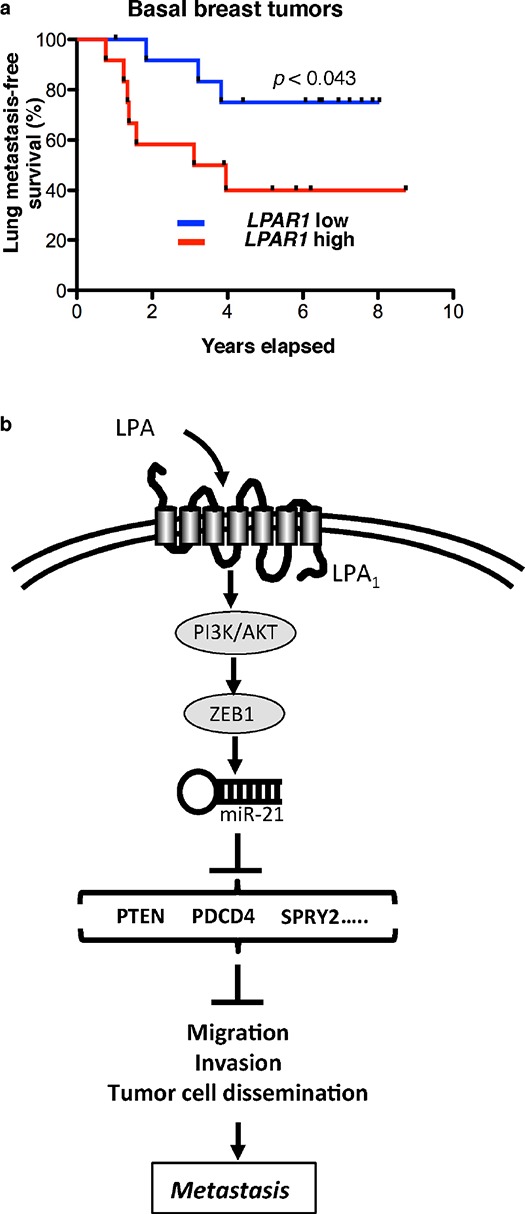
LPAR1 level is a predictive factor for lung metastasis recurrence in basal breast cancers Kaplan-Meier curves depicting 5 year lung metastases-free survival of **a.** 57 non basal breast cancer patients and **b.** 25 patients with basal breast cancer in primary breast tumor database GSE2603. Patients were divided into 2 groups based on the median of expression status of *LPAR1*. In the non basal breast tumor samples *p* < 0.039 versus LPAR1 high group and in the basal breast tumor samples *p* < 0.043 versus *LPAR1* low group using Gehan-Breslow-Wilcoxon test. **c.** Schematic diagram representing the mechanism of LPA-induced early steps of metastasis formation. Acting on LPA_1_ receptor LPA activates PI3K/AKT inducing ZEB1 expression and down-stream activation of miR-21 that by inhibiting the expression of anti-metastatic genes (PTEN, PDCD4, SPRY2) induces cell migration, invasion and metastasis dissemination.

Finally we analyzed whether *LPAR1* levels might have an impact on metastasis recurrence in basal breast cancers. Among several publically available databases with representative breast cancer patient populations sub-classified in basal and non-basal tumors we used the GEO database GSE2603 that additionally provided clinical recording of different tissue metastases. As opposed to records of recurrence data to the lungs the number of bone metastasis events in these populations was too low for developing a comprehensive statistical analysis. However, using the median value of *LPAR1* level as cut-off in each population, the predictive value for lung metastasis-free survival of *LPAR1* levels was significantly different comparing the group of patients with basal tumors versus those with non-basal tumors. Patients with basal tumors but not with non-basal tumors expressing high *LPAR1* levels had a worse lung-metastasis-free survival (*P* < 0.05) (Figure 8a). These results suggested that *LPAR1* level could be considered as a new prognostic factor for patients with basal breast cancers.

## DISCUSSION

LPA exhibits complex mode of actions, due to multi-gene programs activated downstream of multiple LPA receptor subtypes that are frequently co-expressed in cells and tissues and having potential synergistic and opposite functions [1]. Integrating genetic and pharmacological approaches *in vitro* and *in vivo* we demonstrate that the pro-invasive activity of LPA on triple negative breast cancer cells depends on an LPA_1_/PI3K/ZEB1/miR-21 activation cascade.

Overexpression of other LPA receptors, LPA_2_ and LPA_3_, has been previously linked to the progression of breast cancers [4]. However, activated genes driving the oncogenic potential of these receptors have not been defined. As opposed to *LPAR1*, we did not observe significant correlations between *ZEB1* and *LPAR2* or *LPAR3* in publicly available breast cancer cell line databases. The strong correlation between *ZEB1* and *LPAR1* was most prominent in primary tumors of patients with basal breast cancers. Moreover, in addition to a higher prevalence of LPA_1_ expression in basal than in non-basal breast cancer cell lines, there was a significant correlation between *ZEB1* and *LPAR1* in cell line of basal subtypes but not of non-basal subtype. This suggests that controlling ZEB1 expression through LPA_1_ activation might have a functional impact on basal breast cancer behaviors. ZEB1 is a known driver of the epithelial-to-mesenchymal transition (EMT), conferring metastasis and drug resistance properties to epithelial cells and contributing to the poor clinical outcomes [22]. Therefore, mediators of ZEB1 might represent potential therapeutic targets for metastasis suppression. *In vitro*, inhibition of LPA_1_ expression using SiLPA_1_, or blocking its activity with the LPA_1/3_ antagonist Ki16425, significantly inhibited LPA-induced expression of ZEB1. Functional involvement of LPA_1_ in ZEB1 expression *in vivo* was supported by the effect of a short treatment with Ki16425 (5 days) of animals with pre-established skeletal metastases that markedly reduced ZEB1 expression in the tumor metastasis.

LPA_1_ is known to be responsible for LPA-induced cell mobility in both neoplastic and non-neoplastic cells [23]. Here, we demonstrate that specific silencing of LPA_1_ and ZEB1 inhibited the LPA-induced migration and invasion of basal breast cancer cell lines, supporting that ZEB1 is a downstream activated transcription factor of the LPA/LPA_1_ axis stimulating cell motility. Silencing ZEB1 leads to inhibition of bladder cancer cell migration and invasion [24]. In bladder cancer cells ZEB1 regulates vimentin, MMP2 and cytokeratins [24]. Interestingly, MMP2 gene expression correlates to *LPAR1* in human primary breast tumors (hit 23 in Table 1). Moreover, we recently identified a set of 74 early genes including *VIM* (the gene encoding vimentin) that are up-regulated in a LPA_1_-dependent manner in breast and prostate cancer cells stimulated with LPA [25](GEO Series accession number GSE56265). In addition, LPA-induced ZEB1 expression in basal breast cancer cells was mediated through a PI3K-dependent but not of a MEK1-dependent signaling pathway. In bladder cancer cells LY294002, a PI3K inhibitor, inhibits phosphorylation of AKT and GSK3β, and ZEB1 expression due to inhibition of β-catenin/transcription factor 4 (TCF4) complex binding and transcriptional activity on ZEB1 promoter [26]. Intriguingly, our analysis of the nine publically available databases of human primary breast tumors revealed that after ZEB1, TCF4 had the highest Spearman correlation coefficient to LPAR1 (hit 2 in Table 1). Altogether, these data suggest that activation of ZEB1 through the PI3K/AKT pathway might represent a general mechanism in LPA-induced cancer cell invasion and metastasis. In addition, phosphorylation of GSK3β might play a pivotal role in coordinating LPA signals dependent on Pi3K/AKT activation that mediate β-catenin/TCF4-induced ZEB1 expression.

Breast cancer dissemination involves multi-gene programs coordinated by transcription factors. MicroRNAs are post-transcriptional gene expression regulators that are also involved in breast cancer metastasis [27–29]. LPA triggers a series of 74 early genes directly through the activation of LPA_1_ in breast and prostate cancer cells [25]. Here we showed that exposure to LPA for only 45 min up-regulates 36 microRNAs in MDA-MB-231 cells. We focused our attention on miR-21 because it is known to act as an oncomiR. High miR-21 expression is reported to promote invasion and proliferation of multiple cancer cell types, including breast [30, 31] [32–35]. Using a mouse model we confirmed that miR-21 controls breast cancer cell migration and invasion *in vitro*, and metastasis using tumor cell colonization of bone as an endpoint *in vivo*. Moreover, it has been determined that ZEB1 is an upstream regulator of miR-21 [36]. In this study we demonstrated that in human breast tumors there was a significant positive correlation between *LPAR1*, *ZEB1* and miR-21 and that in basal breast cancer cell lines ZEB1 regulates LPA-induced miR-21 expression through an LPA_1_/PI3K dependent mechanism.

We showed previously that blocking LPA_1_ activity inhibits early stage of breast cancer cell metastasis to lungs and bone, independently of cell proliferation and angiogenesis but through inhibition of cell motility and invasion [9]. Inhibiting *in vitro* miR-21 activity with the MirVana miR-21 inhibitor, or silencing LPA_1_ or ZEB1 independently resulted in a complete block of LPA-induced cell migration and invasion. In all cases, basal breast cancer cell functions were rescued by co-transfecting the cells with a miR-21 mimic molecule, indicating that LPA-induced basal breast cancer cell migration and invasion was regulated in a miR-21-dependent manner, down-stream of LPA_1_ and ZEB1 activations. This pathway might have a functional impact on the establishment of metastases as shown by the inhibition of micromedullar metastasis formation with silenced expression of ZEB1 that was rescued following introduction of mirVana miR-21 mimic.

Beside a strong significant correlation between high levels of *LPAR1* mRNA and the lymph node status from an unselected breast cancer population, we previously found that *LPAR1* levels did not predict metastasis recurrence [9]. Recent studies reported that growth of TNBC cells relies upon the pro-inflammatory cytokines IL-6 and IL-8, whose secretion is regulated by the LPA/NF-κB signaling cascade [37]. LPA_1_ is a well-known inducer of IL-6 and IL-8 secretions in ovarian and breast cancer cells [38, 39]. Here, based on the sub-classification of human breast tumors from publically available databases, we found for the first time that as opposed to non-basal breast cancer patients, expression levels of *LPAR1* predicted lung metastasis-free survival in TNBC patients.

Overall our results demonstrate functional relationships between ZEB1 and miR-21 driving LPA-dependent metastasis through LPA_1_ in basal breast cancers (Figure 8c). Our findings may have a major impact by identifying a potential target for development of new adjuvant therapies of triple-negative breast cancer patients to prevent metastatic recurrences.

## MATERIALS AND METHODS

### Cell culture and reagents

Human breast cancer cell lines MDA-MB-231 and Hs578T were obtained and cultured as recommended by American Type Culture Collection (ATCC; Gaithersburg, MD, USA). Characterization of MDA-MB-231/B02 (MDA-MB/B02) and MDA-BO2.Luc breast cancer cells and culture conditions were described previously [40]. Lysophosphatidic acid (1-oleoyl-2-hydroxy-*sn*-glycero-3-phosphate; 18:1) was obtained from Avanti Polar Lipids Inc, Alabama, USA. Ki16425 (LPA_1_/LPA_3_ antagonist) was obtained from Cayman chemicals, Michigan, USA. Wortmannin (PI3K inhibitor) and PD98059 (MEK1 inhibitor) were purchased from TOCRIS, R&D systems.

### Transfection

MDA-MB-231, MDA-BO2, MDA-BO2.Luc and Hs578T cells were transiently transfected with 50nM of mirVana miR-21 inhibitor (50 nM of mirVana miR-21–5p mimic, 50nM mirVana negative control, or 25nM of silencer select pre-designed SiRNA against LPAR1, ZEB1, FOS or STAT3 (all from Ambion, Life technologies), 25nM of BLOCK-iT Alexa fluor-555 as negative control (Invitrogen, Life technologies) using Lipofectamine RNAiMAX Reagent (Invitrogen, Life technologies) following the manufacturer's protocol. All siRNAs were composed of a pool of at least 2 differents siRNAs. The cells were used for *in vitro* or *in vivo* experiments 48 h post transfection.

### RNA extraction, reverse transcription, and real time RT-PCR

Total RNAs including miRNA were extracted using miRNeasy kit (QIAGEN). TaqMan microRNA assays (Applied Biosystems, Life Technologies) were used to quantify miR-21 and RNU-48 levels according to the manufacturer's recommendations. For gene expression analysis, cDNAs were synthesized using iScript cDNA Synthesis kit (Biorad) according to the manufacturer's protocol and real-time quantitative was performed using the SYBR Green PCR kit (Life technologies) using specific PCR primers (Table 2). Each PCR cycle consisted of 10s of denaturation at 95°C, 15s of annealing at 67°C, and 10s of extension at 72°C.

**Table 2:** Primer sequences

Name	Sequence 5′-3′
ZEB1-F	AGCAGTGAAAGAGAAGGGAATGC
ZEB1-R	GGTCCTCTTCAGGTGCCTCAG
STAT3-F	GCCAGAGAGCCAGGAGCA
STAT3-R	ACACAGATAAACTTGGTCTTCAGGTATG
cFOS-F	AGAATCCGAAGGGAAAGGAA
cFOS-R	CTTCTCCTTCAGCAGGTTGG
LPA1-F	AATCGAGAGGCACATTACGG
LPA1-R	CTGTAGAGGGGTGCCATGTT
LPA2-F	CGCTCAGCCTGGTCAAGACT
LPA2-R	TTGCAGGACTCACAGCCTAAAC
LPA3-F	GGAGGACACCCATGAAGCTA
LPA3-R	GGAACCACCTTTTCACATGC
LPA4-F	GCCTGCTACTCTGTCTCAAATTGG
LPA4-R	GCAAGGCACAAGGTGATTGG
LPA5-F	CTCGGTGGTGAGCGTGTACATG
LPA5-R	GCGTAGCGGTCCACGTTGAT
LPA6-F	AAATTGGACGTGCCTTTACG
LPA6-R	TAACCCAAGCACAAACACCA
PDCD4-F	TGGATTAACTGTGCCAACCA
PDCD4-R	TCTCAAATGCCCTTTCATCC
SPRY2-F	CCCCTCTGTCCAGATCCATA
SPRY2-R	CCCAAATCTTCCTTGCTCAG
PTEN-F	ACCAGGACCAGAGGAAACCT
PTEN-R	GCTAGCCTCTGGATTTGACG
L32-F	AGGAGCTGGAAGTGCTGC
L32-R	CAGCTCTTTCCACGATGGC

### miRNA microarray analysis

miRNA from cells were isolated using miRNeasy kit (QIAGEN). Total RNA (100 ng) was directly labeled with Cyanine-3-labeled and array hybridized for 20 h using miRNA complete labeling and hybridization kit (Agilent). The arrays were washed with gene expression wash buffer kit (Agilent) and read with High-resolution microarray scanner (Agilent). The data was extracted using Agilent feature extraction software (Agilent v11.5.1.1). The data were normalized by spike labelling. The median fluorescent intensities were obtained after subtracting background. To identify differential miRNA expression between samples, the median fluorescent intensities were normalized using the median expression values within the array and log2 values analyzed. Data was deposited on Gene expression omnibus, the accession code is GSE64100.

### Western blot analysis

Cells lysates were loaded on a 7% Tris-acetate gel (Invitrogen) and subjected to electrophoresis under reducing conditions. After electrophoresis proteins were transferred onto Immobilon transfer membrane (MerckMillipore) and membranes were incubated with anti-ZEB1 (Abnova), anti-cFOS (Santa Cruz Biotech.), anti-STAT3 (Santa Cruz Biotech.) or anti-α-tubulin (Sigma-Aldrich) antibodies. Detection of bound antibodies was performed using horseradish peroxide (HRP)-conjugated donkey anti-rabbit and anti-mouse secondary antibodies (Amersham; 1/2000 dilution) and with enhanced chemiluminescence detection system (Perkin Elmer LAS Inc).

### Wound healing and invasion assays

Confluent cell culture monolayers were cells scratched using a 10ul tip. Cells were washed with PBS to remove all the debris and incubated for 24 h (MDA-MB-231 cells) or 48 h (Hs578T cells) with serum-free media supplemented with LPA (1 μM). Images were then captured using NIKON Coolpix 990 camera attached to a microscope and the area of cells migrated were tabulated using Morpho-expert software (Explora-nova). Cancer cell invasion across a Matrigel layer was performed as previously described [41]

### Immunohistochemistry

The tumor bearing hind limbs were fixed and embedded in paraffin. Five μm sections were de-paraffinized in methylcyclohexan, hydrated through a graded series of ethanol, then immersed in a peroxidase blocking reagent (DakoCytomation) 5 min. Sections were incubated with normal goat serum for 30 min and incubated overnight at 4°C in humid chambers with primary antibody to Ki67 (dilution1:25) or to human ZEB1 (Abnova, PAB19268, dilution 1:300). The slides were incubated with biotinylated polyclonal rabbit anti-rat immunoglobulin (DakoCytomation) for 45 min. After washing, the slides were treated with peroxidase-conjugated streptavidin (DakoCytomation) for 45 min and developed by addition of a solution of 3, 39-diaminobenzidine tetrahydrochloride (DakoCytomation). Light counterstaining was performed with Mayer's hematoxylin.

### Animal experiments

Mice used in experimental procedures at the Université Claude Bernard Lyon1 (Lyon, France) were handled according to the rules of Décret N° 87–848 du 19/10/1987, Paris. The experimental protocol have been reviewed and approved by the Institutional Animal Care and Use Committee of the Université Claude Bernard Lyon-1 (Lyon, France). BALB/C nude mice purchased from Janvier Labs (Le Genest-Saint-Isle, France), 4 weeks of age, were housed under barrier conditions in laminar flow isolated hoods. Autoclaved water and mouse chow were provided *ad libitum*. Animals were carefully monitored for established signs of distress and discomfort and were euthanized when these were confirmed.

The institutional committee approving the experiments, including details regarding animal welfare are indicated in Supplementary Information 1. For the breast cancer cell colonization to bone (TCB) experiments, transfected MDA-B02.Luc cells were injected intravenously to BALB/c nude mice as previously described [42]. Seven-days post injection, mice were sacrificed and hind limbs were collected, dissected. Bones were minced and treated with 300μL of gentle collagenase/hyaluronidase (Stem cell technologies) for 2 h at 37°C. The cell suspension was washed with phosphate buffer saline (PBS), plated in wells of a 6 well plate in complete culture media supplemented with puromycin (1 μg/mL). After 2 weeks clones of tumor cells that had colonized bone (TCB) were fixed and stained in 50% (v/v) methanol containing 0.05% (w/v) crystal violet and enumerated. For experiments on established osteolytic lesions, animals were injected with MDA-B02.Luc cells as previously described [40] and evidences of metastasis formation were determined by bioluminescence imaging (Nightowl, Berthold, Germany) and X-ray analyses (MX-20; Faxitron X-ray corporation). Metastatic animals were then treated once daily for 5 days with 20 mg/kg Ki16425 or the vehicle.

### Microarray correlation analysis

To identify genes correlated with *LPAR1* we calculated the coefficient of correlation values of *LPAR1* for all genes covered by the given microarray platform across 9 publically available breast tumor datasets (Gene Expression Omnibus (GEO) accession numbers: GSE2109, GSE5460, GSE1456, GSE2034, GSE12276, GSE3494, GSE2603, GSE7390, GSE16391) using R2 genomics analysis and visualization platform. The list of genes in the descending order of coefficient of correlation was generated after averaging the values across all 9 databases. Publicly available gene expression data obtained from three independent studies comprising in total 138 luminal and 75 basal breast carcinoma samples were retrieved from the GEO (accession numbers: GSE20685, GSE21653, and GSE1456). Microarray data were generated on Affymetrix HG-U133Plus2.0 or Affymetrix HGU133A arrays, respectively, and were simultaneously normalized by robust multi-array average (RMA) using custom brainarray CDF files (v17 ENTREZG) yielding one optimized probeset per gene as previously described [43]. *LPAR1* and *ZEB1* expression data from 51 breast cancer cell lines was extracted using BIOGPS online tool from GSE12777 data set. The cell lines were classified as luminal (*n* = 27) and basal (*n* = 24) subtypes.

### Statistical analysis

Analysis of correlation and computation of linear regression of the data were performed using GraphPad Prism v5.0c software. Differences between groups were determined by 1-way ANOVA followed by Bonferroni pos*t*-test. Single comparisons were carried out using non-parametric Mann-Whitney test. Kaplan-Meier analysis for 5 year lung metastases-free survival of 25 patients with basal (triple negative) breast cancer was performed using the GSE2603 dataset. Microarray transcriptomic data was normalized by robust multi-array average (RMA) using R-package yielding one optimized probeset per gene. Patients were divided in 2 groups based on the median of the expression of *LPAR1*. Gehan-Breslow-Wilcoxon test was performed to calculate the *p* value. *p* < 0.05 was considered statistically significant.

## SUPPLEMENTARY FIGURES


