# Supplementary material for: The LPA1/ZEB1/miR-21-activation pathway regulates metastasis in basal breast cancer
Source: Oncotarget. 2015 Apr 27;6(24):20604–20. doi: 10.18632/oncotarget.3774 (PMC4653029; doi:10.18632/oncotarget.3774)
Supplement: Supplementary file 1 [file oncotarget-06-20604-s001.pdf]

SUPPLEMENTARY FIGURES

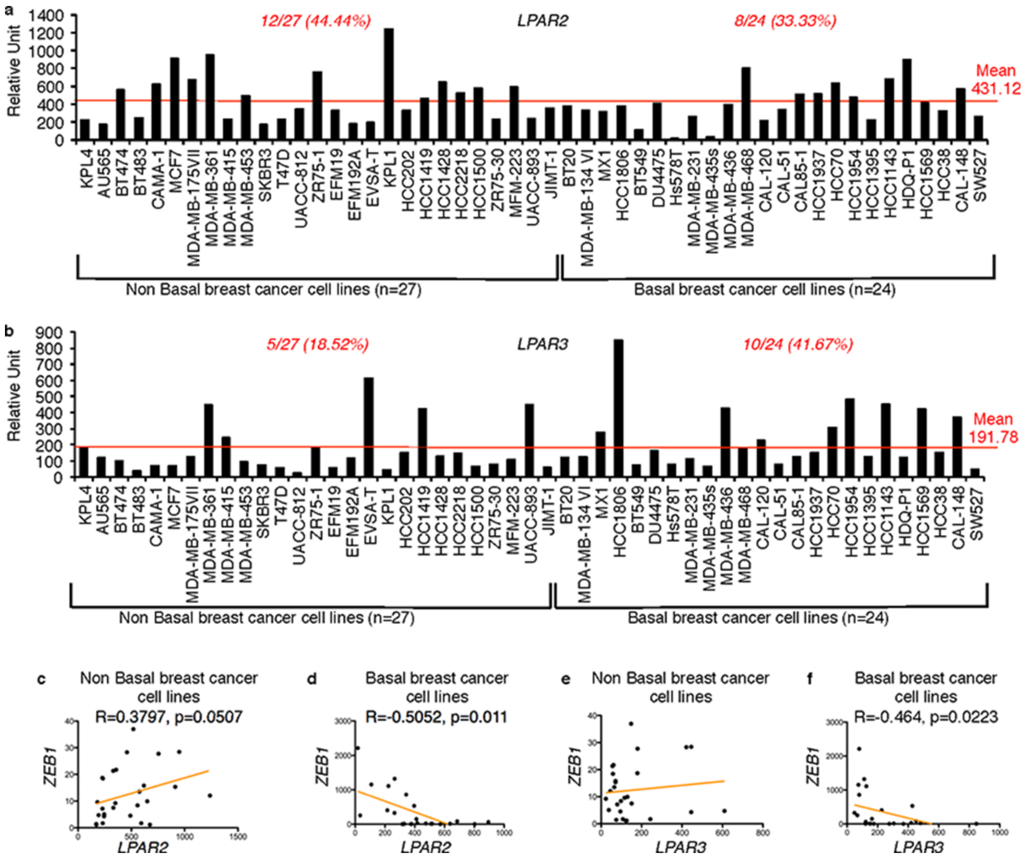

Supplementary Figure S1: *LPAR2* and *LPAR3* do not correlate positively to *ZEB1* in basal breast cancer cell lines. **a.** *LPAR2* and **b.** *LPAR3* expression data from 51 breast cancer cell was extracted using BIOGPS online tool from GSE12777 data set, sub-classified into non basal (n = 27) and basal (n = 24) subtypes. The scatter plots are also shown for the correlation between *LPAR2* and *ZEB1* for both the **c.** non basal (n = 27; r Spearma$n$ = 0.379; p = 0.0507) and **d.** basal (n = 24; r Spearma n = 0.43; p = 0.011) subtypes of human breast cancer cell lines and the correlation between *LPAR3* and *ZEB1* for both the **e.)** non basal (n = 27; r Spearman = −0.020; p = 0.908) and **f.** basal (n = 24; r Spearma$n$ = 0.464; p = 0.0223) subtypes of human breast cancer cell lines.

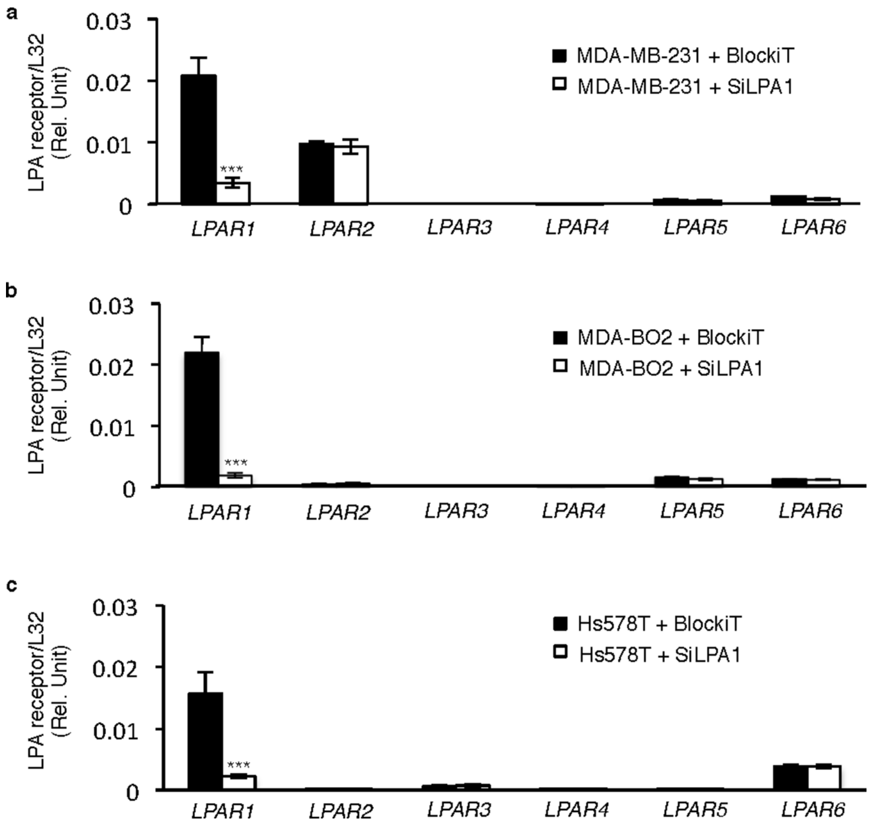

Supplementary Figure S2: Silencing LPA$_1$ expression in basal human breast cancer cells does not alter the expression of the other LPA receptors. Screening of LPA receptors was performed on **a.** MDA-MB-231, **b.** MDA-BO2 and **c.** Hs578T cells transfected with SiLPA$_1$ and BlockiT on LPA stimulation (10 μM) ***, $p < 0.001$ vs BlockIT transfected cells using unpaired two-tailed student *t*-Test. All values were the mean ± SD of 3 experiments.
